# Higher Blood Lactate with Prolongation of Underwater Section in Submaximal Front-Crawl Swimming

**DOI:** 10.3390/sports12050121

**Published:** 2024-04-28

**Authors:** Tomas Venckunas, Justas Achramavicius

**Affiliations:** Institute of Sport Science and Innovations, Lithuanian Sports University, LT-44221 Kaunas, Lithuania; ajustas.achramavicius@ltuswimming.com

**Keywords:** aerobic capacity, anaerobic metabolism, apnea, hypoxia, lactate threshold

## Abstract

The underwater phase (UP) is highly important for overall swimming performance in most swimming events. However, the metabolic effects of the prolonged UP remain unclear. The purpose of this cross-sectional study was to compare the blood lactate response to submaximal front-crawl swimming with short and extended UP. Twelve (four females) junior competitive swimmers (aged 15.4 (1.4) years) undertook 200 m front-crawl swim trials in a 25 m pool at a pre-determined “anaerobic threshold” velocity on two occasions using short (<5 m) and extended (12.5 m) UP after each turn. Pacing and total time were ensured to be identical between the trials. Capillary blood lactate response was measured. Testing for 25 m swim time with <5 m and 12.5 m UP was conducted on a separate occasion. When athletes undertook and extended UP after each propulsion from the wall, their post-exercise blood lactate concentration reached 7.9 (2.1) mmol/L, more than two times higher than the response to trial with short UP (*p* < 0.001). All-out 25 m swimming with <5 m or 12.5 m UP disclosed no difference in locomotion velocity (*p* > 0.05). In conclusion, extending UP of submaximal front-crawl swimming close to maximally allowed during the races substantially increases blood lactate accumulation, i.e., increases the reliance on anaerobic metabolism. Therefore, extended UP is most likely counterproductive for the performance in long-distance swimming, at least for the athletes with a FINA score of <800. On the other hand, the extension of UP could be an effective strategy to train ‘lactate tolerance’, lactate shuttling, removal, and recycling.

## 1. Introduction

Competitive swimmers are ever seeking to improve their performance with training in various aspects, including physiological functions and swimming skills. The latter includes the technique of underwater gliding with undulating body movements after the starting dive and then each turn [1]. In the case of elite swimmers, it has recently been estimated that in a 25 m course pool, turn performance contributes about 50% of the overall race performance (measured as the average speed, i.e., finishing time) across all strokes and distances [2,3]. Undulatory underwater swimming (also known as a dolphin kick) is the fastest way of movement in the water for elite swimmers [1,4], with peak speeds ~15% higher than those of swimming on the water surface [5,6,7]. This difference is multifactorial, but lower wave drag and fusiform shape in undulatory underwater swimming are among the factors that render this way of movement in the water superior to surface swimming in terms of velocity attained [8,9]. The importance of the underwater section for overall swimming performance is also supported by the fact that compared to long-course pools, swimmers are ~2% faster in the short course [10], where the number of turns is higher by two-fold.

According to the current regulations by the International Swimming Federation (Fédération Internationale de Natation, FINA), it is allowed to stay beneath the surface of the water for up to 15 m at the start and after each turn in freestyle, backstroke, and butterfly swimming strokes, independently of the distance of the event and the length of the pool (https://resources.fina.org/fina/document/2024/03/19/e27c972a-b19d-4289-997e-427718461f82/Competition-Regulations-version-1st-January-2024-.pdf, (accessed on 16 April 2024), pp. 76–78). While for most of the high-level competitive swimmers, it is effective to make use of the maximal legal underwater section length in sprint swimming events [2,5,11], taking long underwater phases could not be that beneficial in middle and long distances as it restricts breathing and shortens the period for the effective pulmonary gas exchange. It has been shown in freedivers and synchronized swimmers that longer underwater periods increase blood lactate accumulation in direct relationship with exercise intensity [12]. In support of that, even elite endurance swimmers rarely remain underwater for longer than 5 m after the turns, and the 200 m freestyle performance of elite female swimmers has been shown to be negatively associated with the underwater distances during turns [11]. It could be because of reduced pulmonary oxygen uptake leading to compromised muscle aerobic metabolism during the event [13].

Swimmers implement different strategies for the selection of the underwater phase length after the start and turns, depending on the discipline/event (including swimming pool length), their mastership, physiological and anthropometrical characteristics, and even preferences [6,14]. Sprint swimmers who are capable of high underwater speeds with undulating body movements tend to have longer underwater phases compared to longer-distance swimmers and sprint swimmers who have lower speeds during the underwater phase. It is a common practice of swimmers to restrict breathing during training by using different drills of various breathing patterns during (largely front-crawl) swimming and different horizontal ‘diving’ exercises [15]. In well-trained swimmers, it has been shown that restriction of the number of breathing cycles during submaximal surface swimming decreases arterial blood oxygen saturation and increases carbon dioxide levels and blood lactate concentration [16], although previous similar studies failed to show that blood lactate and heart rate [15,17,18] or expired gases [17] are affected by in-distance (i.e., evenly distributed) restriction of breathing.

To the best of our knowledge, there are no scientifically based recommendations for underwater phase length involving benefit-cost analysis. As aerobic metabolism predominates in energy provision to the active skeletal muscles during swimming for 200 m and above, pulmonary ventilation and gas exchange could play a substantial role in oxygen usage kinetics at the muscular level and, thus, overall performance. However, there are no published studies yet comparing the differences in metabolic response to swimming with different underwater phase lengths. Therefore, the aim of this study was to determine blood lactate response to submaximal front-crawl swimming with intentional modification of underwater phase length in young, highly trained competitive swimmers. Our hypothesis was that lactate response would be significantly greater with a long underwater phase compared to swimming with a short underwater phase.

## 2. Materials and Methods

### 2.1. Subjects

Twelve (four females) highly trained junior swimmers [19] from the same swimming club volunteered to participate in this study. Inclusion criteria were the following: age 18 years or younger; at least 6 years of training experience in swimming; competitive level—personal best of at least 500 FINA points; and training volume of at least 1500 km during the year preceding the study. Exclusion criteria were any recent disease or injury that could affect swimming performance. Participants were trained by the same team of four professional swimming coaches and were part of the national talent development system. Swimmers were training for a total of ~28 h per week, which included 40 to 70 km of swimming per week on a regular basis, and had achieved national to international competitive level at the time of investigation.

The numerical characteristics of the participants are presented in Table 1. Swimmers had been using in their training the technique of various strokes and were about equally representing all competitive swimming strokes (except for backstroke) and distances in which they were specializing the last season and had the best FINA score (Table 1).

### 2.2. Study Design

This cross-sectional study was conducted at a home training facility’s short-course (25 m) indoor swimming pool. The water temperature was maintained at 27.5 °C and room temperature at 27 °C during all four days of the investigation. All swimmers conducted a standardized warm-up of ~30 min, comprising 1000 to 1400 m of swimming at a self-selected pace using different strokes and drills, not exceeding ~140 bpm, and some light land-based drills of their usual warm-up routine. This study was approved by a local ethics committee, and before being enrolled in this study, all participants read and signed the informed consent form.

On the first day of the investigation, an increasing intensity step test was conducted to determine the required pacing for the subsequent 200 m swimming bouts. The step test was conducted within 2 weeks before the first 200 m swimming bout, which was aimed at the pace corresponding to 4 mmol/L capillary blood lactate concentration determined during a step test. In a randomized order, during separate training sessions, the subjects then swam the 200 m front-crawl bout twice, with their usual 4–5 m underwater section after each turn and with the half swimming pool length (i.e., 12.5 m) underwater phase. These two 200 m bouts were separated by 7 days, during which the standard training was performed, and both were held after the day of rest. The coach was hand-signaling on the reduction or acceleration in swimming speed after each 25 m if required.

During all of the tasks, athletes wore the same personal goggles and attire, which met the eligibility requirements for swimming competitions according to the existing regulations. The same experienced researcher used an electronic handheld stopwatch to control for swimming speed each 25 m and to record each 50 m split.

### 2.3. Incremental Intensity Step Test

Subjects performed a slightly modified version of the widely used 7 × 200 m incremental swimming test developed by the Australian Institute of Sports [20,21]. In brief, seven constant-speed front-crawl swimming bouts of 200 m were repeated on a 5 min cycle, graded from easy to maximal. The target times for each step were individualized: the last step was aimed at 5 s slower than the personal best result for the 200 m front crawl, and each previous step was targeted to be 5 s slower than the one after. Thus, swimming times between the slowest (first) and the fastest (last, seventh) steps were aimed to be different by 30 s for each athlete, and the seventh (final) swim was performed with maximal efforts [21,22]. Each step was initiated in the water with a push start from the wall. The subjects corrected their speed during the test according to the visual signals of the coach. The time was recorded by a stopwatch, and capillary blood lactate concentration (fingertip) was measured before the test and 1 min after each stage. The second-degree polynomial trendline of the relationship between swimming speed and blood lactate concentration was generated using Microsoft Excel. The resultant speed corresponding to 4 mmol/L blood lactate, which is approximately the average lactate value at the anaerobic threshold for a front crawl in elite swimmers [23], was a target pace for the 200 m swims with the short and prolonged underwater sections (performed on separate days).

### 2.4. 200 m Front-Crawl Swimming Tasks

The swimmers started from the water at the start area of the pool by deciding to start themselves when ready. The target pace was determined as described above to correspond to 4 mmol/L capillary blood lactate concentration determined during a step test with normal convenient ad libitum breathing. The subjects swam in one of the middle lanes and conducted the task, individually paced by the coach if adjustments in pace were required. However, this was rarely necessary since the athletes were experienced enough to pace themselves largely without much deviation from the schedule. All athletes adopted their usual freestyle swimming technique (stroke length, breathing pattern, ratio of the involvement of the arms and legs, leg kick frequency, etc.) without additional instructions, except for the prolonged underwater sections condition, where they were required to propagate themselves underwater using the dolphin kick with their arms extended forwards (and palms folded together in the horizontal plane) up to the middle of the pool (i.e., for 12.5 m) after each turn. A flamboyant floating foam pipe was fixed to the wires separating lanes perpendicular to the swimming direction exactly in the middle of the swimming pool, and subjects were instructed to emerge onto the surface of the water immediately after crossing this marking. The turns were performed by doing half somersaults.

### 2.5. Maximal Speed 25 m Swimming Tasks

Within the next two weeks after the second 200 m trial, swimmers underwent two 25 m all-out front-crawl swimming trials, one with a 4–5 m underwater phase at the start and another with a 12.5 m underwater phase. These were performed after a standardized warm-up (the same as preceding the 200 m trials) in a randomized order with ~10 min of rest in between to allow for a full recovery after the first trial. Swimmers were allowed for easy activity at their disposition in or outside the water during this recovery period. The main task of the swimmers was to complete the 25 m section as fast as they could. The swimming was conducted individually (one subject at a time) from the water (by pushing off a wall) in one of the middle lanes. Athletes themselves decided when to start and adopted their usual freestyle swimming technique (stroke length, breathing pattern, ratio of the involvement of the arms and legs, leg kick frequency, etc.) without additional instructions for style except for the underwater section length. For the 12.5 m underwater phase, a piece of floating foam was fixed to the lane separators exactly in the middle of the pool, and subjects were instructed to emerge onto the surface of the water immediately after crossing this marking.

### 2.6. Measurements

Mixed capillary blood lactate concentration was measured from the fingertip using a commercially available portable analyzer, Lactate Pro 2 (Arkray, Kyoto, Japan). A single reagent strip has been used per time point of measurement, which absorbs 0.3 µL of blood volume via a capillary principle and provides a reading for lactate concentration (in mmol/L) within 15 s. Lactate was first measured a few minutes after the completion of warm-up before the start of the swimming bout. The eligibility to start the test or task was lactate concentration below 3 mmol/L. Lactate was also measured within 1 min after each of the 200 m in the incremental intensity step test and 1 min and 5 min after the completion of the 200 m front-crawl swim bout with short or prolonged underwater sections.

### 2.7. Statistics

Since there were no noticeable differences in the main outcome measures between the gender groups, we presented the data as means and standard deviations for the combined group. Paired Student’s *t*-test was used to compare the means between conditions and between different time points. The level of significance was set at *p* < 0.05.

## 3. Results

Swimmers managed to perform both tasks, the one with unrestricted comfortable breathing and another one with prolonged underwater sections, at nearly identical final times and pacing (Table 2). Baseline lactate values were the same before the two 200 m swimming tasks, while the increase was much more pronounced when swimmers were undertaking the long underwater phase strategy (Table 2).

The maximal swimming speed test revealed the times for either the 12.5 m (half swimming pool length) or 25 m were not significantly different with a prolonged (12.5 m) diving compared with a short (<5 m) one. However, both the 12.5 m split time and the whole 25 m segment time tended to be slower (*p* = 0.063 and *p* = 0.066) when a long underwater phase was implemented (Table 3).

## 4. Discussion

The aim of this study was to investigate blood lactate response to submaximal front-crawl swimming by modifying the underwater phase length in young, highly trained competitive swimmers. The results of this study have shown an exaggerated blood lactate response to the bout of submaximal swimming when performed with an extended underwater section after each turn. When athletes, instead of their comfortable breath-hold duration at each turn, undertook long (12.5 m) underwater sections after each propulsion from the wall in a 25 m pool, their blood lactate level was more than doubled, and the delta (accumulated) blood lactate increased even to a larger extent. This increased reliance on less efficient anaerobic metabolism would be unfavorable for the performance in endurance swimming events, but it could be effectively used for specific training adaptation purposes.

For the majority of our subjects, the task with extended underwater sections was subjectively much more demanding (personal communication with the athletes) despite the extensive practice of different types of breath holds and undulating underwater swimming as a substantial component of their athletic routine consisting of an average of 4 h of training per day. Indeed, even if the speed of the swimming task was individually adjusted for blood lactate not to exceed 4 mmol/L when using a normal breathing pattern (i.e., taking short underwater sections), this was not the case with prolonged underwater sections, where the lactate levels soared to nearly 8 mmol/L on average. The latter modality of swimming could not have been maintained for much longer than 200 m of continuous swimming, as admitted by most of the athletes themselves. That is, the task with long underwater phases has proved to be performed at an intensity clearly above the lactate levels corresponding to the so-called lactate (anaerobic) threshold.

Swimmers of a similar caliber to those in this study that were investigated for the effects of three different lengths of underwater sections during the 2 × 75 m trials of 200 m competitive pace reported higher perceived exertion with extended underwater sections, and even though their lactate levels and heart rates tended to be higher with extended underwater sections, this did not reach statistical significance [24]. The seeming discrepancy between the results of our study and the outcomes of the study by Veiga et al. [24] is most likely due to the intensities used: when swimming is performed at a 200 m competitive pace, lactate production and appearance in the blood approach peak values even with less restricted breathing using short underwater sections after each turn. The increased rate of both aerobic and anaerobic metabolism occurring at an intensity above the lactate (ventilatory) threshold could compromise the swimmers’ ability in the long underwater sections and increase perceived exertion. However, intervals with long underwater sections after each turn can be repeated multiple times if in-between rest for, e.g., a few minutes was allowed. Also, to compensate for the need for much increased pulmonary ventilation at high intensities of prolonged intervals, stroke frequency increases [25], allowing a swimmer to effectively increase breathing rate.

Since the aerobic metabolism within the working muscles is activated close to maximally during ~2 min of all-out tasks such as 200 m freestyle swimming [26], limiting oxygen provision and thus aerobic metabolism by, e.g., restricted breathing via prolonged underwater sections could be counterproductive for the overall performance. It is also interesting that the underwater section after the turns during 100 m and 200 m freestyle at the very elite level is the shortest compared to that of other strokes [11,27], and that is the case despite the front crawl being the fastest stroke. Consequently, even shorter time is spent in front crawl after each turn compared to breaststroke and butterfly strokes, while it should be acknowledged that breath-hold in front crawl (and backstroke) starts somewhat earlier before the push-off from the side of the pool since the rules do not require to touch the wall with hands and therefore swimmers perform half somersaults to make the turns faster and receive the most benefit from the plyometric action of the legs in terms of velocity [3]. The proof of efficacious mastering of the turns at the elite level is ~2% faster competitive times in a 25 m pool compared to a 50 m swimming pool [10].

Our results suggest that competitive swimming beyond 200 m with a 10–15 m underwater section after each turn in a 25 m pool would most likely be not feasible for sub-elite athletes at least, or highly specific preparation would be required. More than that, for most of the swimmers at this level, this mode would not be beneficial in terms of performance in any distance since a tendency for a slower 25 m time trial with a prolonged underwater phase. Therefore, the extended underwater section does not seem to be useful for performance beyond the sprint distances, at least until the propulsion using dolphin kick is perfected to outweigh the diminished oxygen availability and reduced capacity for aerobic metabolism with a higher speed under the water surface. This is somewhat in the analogy of the balancing effects of high-altitude rarified air with its reduced resistance (“ergogenic” effect) but also hypoxic environment (“ergolytic” effect) on endurance cycling or sprint running performance.

No difference in underwater section distance for 100 m and 200 m freestyle performance between national- and regional-level swimmers has been reported despite underwater section distances of higher level athletes being longer in other strokes [27]. This may suggest that there is still room for improvement in freestyle performance in even well-trained swimmers if they adopt longer underwater sections during competitions. However, apart from metabolic constraints, the long underwater phase strategy is challenging [24], as holding the breath for a large fraction of the distance requires good mental resilience, a quality that is both malleable by restricted breathing during workouts and important for the overall competitive swimming performance. Different modes of breathing restriction training are vastly implemented in the training of swimmers [15]. For instance, apneic sprint training has been shown to effectively increase 100–400 m swim performance, the effect attributed largely to upregulated anaerobic glycolytic metabolism [28]. There is evidence to suggest that repeated exposure to underwater apneic exercise reduces lactate production in response to apneic exercise [29], probably also evidencing specificity in adaptation and warranting a progressive increase in the stimulus (more repetitions, shorter rest periods, etc.).

There is strong evidence to suggest that respiratory (pulmonary) function may limit endurance performance even in unrestricted sea-level environments [30], and this may be of particular relevance in sports such as swimming, where pulmonary ventilation is restrained by the sports-specific requirements or environment. In collegiate swimmers, reduced breathing frequency during submaximal 200 m swim has been shown to induce greater inspiratory muscle fatigue and larger lactate accumulation compared with unrestricted breathing [31], and inspiratory muscle fatigue has been shown to reduce middle distance swimming performance [32]. Similarly, exercise-induced bronchoconstriction in response to stair races is associated with slower finishing times, putatively due to premature fatigue caused by impaired delivery of ambient oxygen to the exercising muscles [33]. Competitive swimmers are particularly prone to exercise-induced bronchoconstriction [34], and prolonged underwater sections for those affected would reduce the “window” for pulmonary ventilation during the training and/or competitions. However, it remains to be tested whether exercise-induced bronchoconstriction could be reduced by swimming with longer underwater sections and consequently with relative sum hypoventilation and resultant hypercapnia. There is evidence that one of the main factors of exercise-induced bronchoconstriction is hyperventilation [35], while carbon dioxide has a direct relaxant effect on the airways [36].

Higher lactate levels in response to swimming with prolonged underwater sections reported in this study could putatively be because the underwater propulsion phase is realized largely by lower body muscles, while for surface front-crawl swimming, upper body muscles are the primary driving force. However, we feel that this could barely be the explanation for exaggerated blood lactate response since although leg muscles are more bulky and perhaps not that efficient for swimming, they are also trained during swimmers’ training routines [28] and are not more anaerobic compared to arm muscles [37]. In addition, the large increase in lactate levels with prolonged underwater sections would not be attributable to the intensity of the contractile activity of the muscles per se since the task was submaximal rather than maximal. On another end, in top-level swimmers, blood lactate levels have been shown to soar impressively with all-out swimming bursts of as little as 10–15 m [38], which was actually above the levels reported after swimming races of any distance and stroke [39].

We propose that the restriction of breathing through the extension of underwater phases after each turn could be an effective strategy to train ‘lactate tolerance’, lactate shuttling, removal, and recycling, including more efficient usage of the Cori cycle [40]. It could be further speculated that upregulated lactate production and elevated levels in the blood could additionally nourish and promote the gut microbiome to aid both health and athletic performance [41]. Unequivocally, increasing the volume of underwater swimming during training would also be expected to help master gliding and undulatory swimming, thus making the underwater section faster and promoting overall distance swimming performance.

In this study, the breath-hold duration with extended underwater phases comprised >1/2 of the total 200 m swimming task time when calculating breath-holds before the turns when still on the surface, then the somersault tuck, and finally the undulatory swimming underwater after the propulsion of the wall. In a 50 m pool, this “duty cycle” (underwater to surface swimming distance ratio) would maximally be ~1/3 even when the full 15 m legal underwater sections are undertaken. Thus, the effects of the extension of underwater phase distance or duration in a 50 m long pool on metabolic demands are left to be investigated.

## 5. Conclusions

A more than doubled blood lactate concentration was achieved during submaximal front-crawl swimming in the 25 m pool when extending the underwater section close to that which is still legal during the races. This tactic of prolonging the underwater section would seemingly not enhance performance beyond the sprint distances, at least until the underwater swimming with a dolphin kick is mastered sufficiently to receive the benefits of higher speed to outweigh hindered aerobic metabolism. However, the extension of the underwater phase during training could be an effective strategy to augment training adaptation by repeatedly inducing a temporal ‘oxygen debt’ and not only training the lactate metabolism but possibly also augmenting the adaptations of aerobic capacity within the trained muscles. Specifically for the swimmers, augmenting the performance with individually adjusted underwater section length for different distances would require further research.

## Figures and Tables

**Table 1:** Characteristics of the participants (n = 12).

	Mean (SD)	Range
Age, years	15.4 (1.4)	13–18
Training experience, years	7.8 (1.2)	6–10
Body height, cm	179.1 (8.4)	166–194
Body weight, kg	66.8 (9.3)	52–80
Body mass index, kg/m^2^	20.7 (1.9)	18.1–24.2
Personal best FINA score	636 (86)	530–789
***Specialization***		
Freestyle, 50–100 m	2 males, 2 females	
Freestyle, 200–1500 m	3 males	
Breaststroke, 100–200 m	1 male, 1 female	
Butterfly, 50–100 m	1 male	
Medley, 100–200 m	1 male, 1 female	

**Table 2:** Blood lactate concentration in response to paced 200 m front-crawl swimming bout in highly trained junior swimmers (n = 12) using short and long underwater diving.

	Short (<5 m) Underwater Section	Long (12.5 m) Underwater Section
Lactate at baseline, mmol/L	2.0 (0.5)	2.4 (0.6)
Lactate 1 min post, mmol/L	3.3 (1.4) ^#^	7.9 (2.1) * ^#^
Lactate 5 min post, mmol/L	3.0 (1.2) ^#^	6.6 (1.8) * ^#^ ^
Lactate accumulation, mmol/L	1.3 (1.0)	5.6 (2.3) *
200 m swimming time, s	154.5 (12.7)	156.0 (12.9)
1st 50m split, s	37.2 (3.1)	37.6 (3.3)
2nd 50m split, s	38.5 (3.4)	39.1 (3.5)
3rd 50m split, s	39.5 (3.2)	39.9 (3.3)
4th 50m split, s	39.3 (3.5)	39.4 (3.7)

* Difference between the conditions at *p* < 0.0001. ^#^ Difference from baseline at *p* < 0.0001. ^ Difference from lactate at 1 min post, *p* = 0.002.

**Table 3:** Maximal swimming speeds of highly trained junior swimmers (n = 12) using short and long underwater diving.

	Short (<5 m) Underwater Phase	Long (12.5 m) Underwater Phase
12.5 m time, s	6.00 (0.44)	6.54 (0.85)
25 m time, s	13.13 (0.86)	13.51 (1.15)

## Data Availability

Data is contained within the article.
